# Role of Microglia in Neuropathic Pain

**DOI:** 10.7759/cureus.43555

**Published:** 2023-08-16

**Authors:** Miltiades Y Karavis, Ioanna Siafaka, Athina Vadalouca, George Georgoudis

**Affiliations:** 1 Musculoskeletal Physiotherapy Research Laboratory, Department of Physiotherapy, University of West Attica, Athens, GRC; 2 1st Department of Anesthesiology, National and Kapodistrian University of Athens School of Medicine, Athens, GRC

**Keywords:** spinal cord, neuroinflammation, neuropathic pain, microglial cells, microglia

## Abstract

Microglial cells are specialized macrophage cells of the central nervous system responsible for the innate immunity of the spinal cord and the brain. They protect the brain and spinal cord from invaders, microbes, demyelination, trauma and remove defective cells and neurons. For immune protection, microglial cells possess a significant number of receptors and chemical mediators that allow them to communicate rapidly and specifically with all cells of the nervous tissue. The contribution of microglia in neuropathic pain challenges conventional concepts toward neurons being the only structure responsible for the pathophysiological changes that drive neuropathic pain. The present study is a narrative review focusing on the literature concerning the complex interaction between neurons and microglia in the development of neuropathic pain. Injury in the peripheral or central nervous system may result in maladaptive changes in neurons and microglial cells. In neuropathic pain, microglial cells have an important role in initiating and maintenance of pain and inflammation. The interaction between neural and microglial cells has been proven extremely crucial for chronic pain. The study of individual mechanisms at the level of the spinal cord and the brain is an interesting and groundbreaking research challenge. Elucidation of the mechanisms by which neurons and immune cells interact, could constitute microglial cells a new therapeutic target for the treatment of neuropathic pain.

## Introduction and background

Neuropathic pain is defined by the International Association for the Study of Pain as “pain caused by a lesion or a disease of the somatosensory nervous system.” Unlike nociceptive pain, which is the normal response to noxious stimuli, neuropathic pain is a maladaptive response to an injury of the nervous system [1]. In neuropathic pain, which can be characterized as acute or chronic and central or peripheral, tissue damage is not always obvious. The most common causes of neuropathic pain include trigeminal neuralgia (idiopathic), post-amputation pain, persistent post-operative or post-traumatic pain, post-herpetic neuralgia after a herpes zoster infection, painful diabetic polyneuropathy, pain in patients with cancer and those infected by HIV, and pain as a side effect of certain medications (chemotherapy) [2]. As pain is a subjective measurement, diagnosis of neuropathic pain is difficult. Common features of neuropathic pain are the presence of allodynia, where a normally non-painful stimulus results in pain, and hyperalgesia, where there is an increased response to a painful stimulus, and spontaneous pain [3].

Regardless of etiology, location, intensity, or other individual characteristics, neuropathic pain is characterized by changes in the normal processing of sensory signals. Despite the absence of tissue damage, the pain is maintained through the development of peripheral and central maladaptive mechanisms. The disorder develops gradually for weeks or months after damage, decisively affecting patients’ quality of life. Once neuropathic pain is generated, the sensory hypersensitivity typically persists for prolonged periods, even though the original etiological cause may have long since disappeared, as after nerve trauma. Symptom persistence ultimately causes changes in the genetic expression of the neurons of the somatosensory system and extension of the cortical representation in areas of the primary sensory cortex [1]. First-line treatment modalities for neuropathic pain do not target the underlying mechanisms causing the pain and are not specific to the different phases in the development of neuropathic pain [4,5].

Microglia, first described in 1920 by del Río Hortega, are a specialized population of mononuclear macrophages that are located in the central nervous system (CNS) and comprise ~10-15% of all glial cells. They participate in immune surveillance and homeostatic control of inflammation and regulate the innate immune response of the brain and spinal cord. They develop from the progenitor hematopoietic stem cells (myeloid progenitor cells) during early embryogenesis and migrate initially to the brain in relatively small numbers [6]. However, their impressive proliferation ability allows them to rapidly colonize the entire CNS. This ability is maintained eternally through the colony-stimulating factor 1 (CSF-1), which is expressed in the membrane of microglial cells in the adult brain and is associated with the expansion of the microglia population during neuroinflammation and neuropathic pain [7].

Microglial cells are considered as the fastest and most flexible cells in the brain. They have a small cell body and several long processes with multiple branches that allow them to monitor each neuron individually while "slipping" among them by extending and shrinking their branches [8]. They are oriented in space and are attracted by ischemia or brain damage. They constantly control all spinal and cerebral regions, the synapses of each neuron, and each neural circuit [9]. They stay in touch with each of the synapses for a few minutes and then move on to the next one. They continuously evaluate the usefulness of the neural circuits, prioritizing frequently used synapses and completing the scanning of billions of cerebral and spinal synapses within a few hours. This constant vigilance of microglial cells protects the brain and spinal cord from invaders, germs, demyelination, trauma, and mutated or defective cells [10]. Microglia respond quickly with morphological changes when stimuli affect the physiological homeostasis of the CNS: microglia numbers increase, volume increases, processes retract and become ramified. Activated microglia have two polarization types: M1-type (M1) and M2-type (M2), pro-inflammatory and anti-inflammatory, respectively. M1 microglia have a strong phagocytic ability and can produce many pro-inflammatory factors [11].

The present study is a narrative review focusing on the literature concerning the complex interaction between neurons and microglia in the development of neuropathic pain.

## Review

Following a peripheral nerve injury, the system of pain management and treatment malfunctions. Research efforts and pharmacological approaches have focused on neural tissue dysfunction. However, evidence suggests that in addition to nerve cells, specialized non-neural cells that regulate the innate immune response of neural tissue against pathogens, play an important role in this dysfunction, which ultimately leads to neuropathic pain [12-14]. An increase in the number of microglial cells in the spinal cord after peripheral nerve damage has been reported since 1970 [15], and perhaps this action of glial cells is the missing element to explain the conversion of acute pain to chronic [16,17].

Laboratory experiments using modern tools of molecular genetics have shown that microglia participate in the onset, maintenance, mediation, and expansion of neuropathic pain [13,18]. In models of neuropathic pain (compression of the peripheral or spinal nerve), inhibition of microglial cell activity led to milder pain. Advanced research techniques (transgenic and chimeric animal models of gene targeting) have shown that nerve damage activates microglia causing rapid changes in morphology, migration rate, and proliferation [19]. A recent study used chemogenetic approach as a novel tool to examine the role of microglia in neuropathic pain in mice. Researchers used transgenic mice and demonstrated that microglial Gi signaling by chemogenetic manipulation attenuates chronic pain via inhibition of neuroinflammation [20]. An increase in the number of protein molecules on the surface of microglia (more than 40 have been identified) after peripheral nerve damage, indicates that microglia is one of the contributors to neuropathic pain [11,21].

Neuroglial cells (microglia, astrocytes, oligodendrocytes, and ependymal cells), besides the support and nutrition of neurons, actively participate in the reception, transmission, processing, and modification of nerve stimuli. Glial cells and neurons closely cooperate aiming at homeostasis and optimal functioning of the sensory synapses. In pain and tissue damage, neurons are assisted by glial cells that are activated at the dorsal root ganglia and the posterior horns of the spinal cord. Glial cells are co-responsible to some extent for the expansion of pain. Many non-opioid and opioid peptides act on microglial cells, which respond by producing pro- or anti-inflammatory agents to a continuous supplementation of neurons with excitatory chemical signals [22,23].

From the peripheral nerves to the spinal cord

In nerve damage (trauma, neuropathy, surgery, etc.), inflammatory irritants fill the space around the free nerve endings, peripheral receptors, axons, or peripheral stumps. The sensory nerves, through their special channels, including transient receptor potential vanilloid 1 (TRPV1), prostaglandin receptors, tendon-sensitive ion channels, etc.), convert chemical stimuli into electrical energy potentials. Glial cells around the spinal synapses respond to both electrical and chemical stimuli; tensile cells in the cell membrane "sense" the intensity of nerve activity and respond to it with hyperstimulation. Astrocytes and microglial cells, which are scattered but regularly arranged throughout the CNS and along the somatosensory pathway, express receptors for most of the substances that act on the spinal sensory synapsis. On the membrane of glial cells, there are glutamic acid receptors (ionotropic: N-methyl-D-aspartate or NMDA and α-amino-3-hydroxy-5-methylisoxazole-4-propionic acid or AMPA; metabotropic: mGluR I, II, III), gamma-aminobutyric acid receptors (GABA A & GABA B), norepinephrine receptors (α1, α2, β1, and β2 inactivated protein), acetylcholine receptors (nicotinic α3, α5, α6, α7, and β4 receptors), cannabinoid receptors (CB2) and receptors of bradykinin, dopamine (D1 & D2), purines, adenosine, and opioids [24].

Some transmembrane receptors are expressed in resting microglia and others in the activated state. Toll-like receptor 4 is overexpressed in microglial cells after their activation. Its overexpression promotes the production of nitric oxides, prostaglandins, leukotrienes, nerve growth factors, stimulant amino acids, neurotoxic peroxides, and cytokines, such as interleukin-1 (IL-1), interleukin-6 (IL-6) and tumor necrosis factor-α (TNF-α). These substances further stimulate the perisynaptic region in the microglia-axons positive feedback loop. This toxic compound of chemical substances originating from nerve cells, astrocytes, and microglial cells is responsible for chronic and persistent neuroinflammation [25-27].

Evidence has led to the hypothesis of "microgliopathic pain," a pain syndrome for which dysfunctional microglia are responsible. It has been thought that the theory of dysfunctional microglia in chronic pain may be the "missing link" in pain physiology. Dysregulated microglial activation, neuroinflammation, and altered neuronal communication contribute to the amplification of pain signals and the transition from acute to chronic pain. By targeting microglial-related mechanisms, researchers are striving to develop effective treatments that can provide relief to individuals suffering from chronic pain [28].

Events in the spinal cord

After peripheral nerve damage, the resting microglia of the spinal cord rapidly changes both morphologically and functionally. Resting microglial cells have small cell bodies and long-branched processes. As microglial cells are activated, the cells undergo morphological changes in which the cell body becomes relatively large, and the processes become shorter. These changes are accompanied by an increase in the number of microglia in the spinal cord, either by migration or proliferation, a phenomenon known as gliosis or microgliosis [29]. These activated microglial cells, also called amoeboid microglia, have higher rates of migration, proliferation, and phagocytic behavior. Besides the morphological changes in microglial phenotype, an increase in surface immunogenic antigens and the production of cytotoxic and neurotrophic factors has been reported [30-32].

Besides cellular hypertrophy, overexpression of the complement receptor CR3 on the microglial cell surface is presented, changes that are observed within the first 24 hours of nerve damage. Two to three days after nerve injury, microglia begin to proliferate, reaching the maximum number of cells on the 4th to 7th day. Within the first seven days, the most prominent changes in the immunophenotype are observed, with more frequent increased expression of major histocompatibility complex (MHC) antigens. The changes are reversible regardless of the severity of the damage and usually are over one month after the start of the damage. Temporal continuity was one of the reasons for accepting the role of microglia in the structural changes of sensory cells and in the genesis of neuropathic pain [33-36].

CSF-1 has been identified as the key chemoattractant of microglial cells, which invites them to colonize and expand at the site of the lesion. Concurrently, its receptor, CSF1R, located on the surface of glial cells of the spinal cord, increases in number proportionally [37]. It simultaneously interacts with a) DNAX-activating protein 12 (DAP12), a transmembrane signal transduction protein responsible for microglial cell proliferation, and b) a second DAP12-dependent signaling pathway through which some specific microglial genes associated with pain sensitivity are up-regulated, such as interferon regulating factor 8 (IRF8) and 5 (IRF5). The expression of IRF8 after peripheral nerve injury activates IRF5. IRF5 specifically and selectively binds to the promoter of the purinergic receptor P2X 4 (P2RX4) gene encoding the receptor P2X4R, which is expressed on the cell membrane of microglia [36,38-41]. Activated P2X4 receptors bind adenosine triphosphate (ATP) and lead to signaling (activation) of other pro-inflammatory pathways and maintenance of neuropathic pain [40,42,43]. CSF-1 is now considered one of the factors that end up in the spinal cord and are associated with the activation of microglia. Both CSF-1 and DAP12 are important pharmacologic targets.

The fractalkine receptor, C-X3-C motif chemokine receptor 1 (CX3CR1), participates in microglial activation as well. This receptor is expressed on the cell membrane of microglial cells of the spinal cord and brain, it binds to fractalkine (chemokine-adhesion molecule) and is overexpressed in the spinal cord during inflammation and nerve damage [44,45]. The binding of fractalkine to its receptor (CX3CR1) initiates the p38/MAPK serine/threonine protein kinase intracellular signaling pathway. This is considered the most basic signaling pathway inside microglia that controls cell proliferation, cell response to stress, cell death, differentiation, migration, etc. It is associated with the synthesis and release of a wide variety of cellular mediators of inflammation, such as brain-derived neurotrophic factor (BDNF), TNF-α, IL-1b, and IL-6 by microglia, which, when secreted, enhance synaptic transmission and stimulate interstitial facilitatory neurons [41,46-48].

Summarizing, three main elements could be reported in the mechanism of activation of the spinal microglia. First, nerve damage leads to de novo CSF-1 induction in the dorsal root ganglion. From here, CSF-1 reaches the ends of the sensory neuron (reciprocating movement) and recruits the CSF-1 receptors (CSF-1Rs). Second, the presence of CSF-1 is considered a capable and necessary condition for the proliferation, migration, and activation of microglia. Finally, the activation simultaneously affects the entire reflex arc, the sensory neurons of the dorsal horn, the medial neurons, and the corresponding anterior horn motor neurons. The purine (ATP) and fractalkine pathways are rather involved in the maintenance and perpetuation of pain than in the initial activation and sensitization.

Activation of the spinal neuroglia is observed in the initial phase (onset phase) of neuropathic pain and precedes the activation of astrocytes (astrogliosis), supporting the hypothesis that microglia contribute to the initiation of neuropathic pain while astrocytes participate in its maintenance [49,50]. There is considerable evidence that microglial activation is both a sufficient and necessary condition to produce central sensitization and neuropathic pain [51,52].

Events in the brain

The role of neurotransmitters in the function of microglia has been examined in the brain. Neuropeptide Y (NPY), vasoactive intestinal polypeptide (VIP), pituitary adenylate cyclase-activating peptide (PACAP), somatostatin, cortistatin, tachykinins, calcitonin gene-related peptide (CGRP), leptin, ghrelin, and neuropeptides derived from the POMC gene (proopiomelanocortin - hypothalamic nucleus toxoid), such as adenocorticotropin-releasing hormone (ACTH), melanocyte-stimulating hormone (MSH), and opioid peptides (μ, δ, and κ receptors) directly affect the function of microglial cells. Microglial cells express membrane receptors for all the above-mentioned neuropeptides, thus participating in the regulation of pain sensitivity [53,54].

The role of brain microglia in chronic neuropathic pain also emerged from animal studies [55,56]. It was found that peripheral nerve damage led to significant microglial activation (microgliosis) in the thalamus, somatosensory cortex, and parietal system, brain areas associated with chronic pain, and the emotional and cognitive response to it [57]. The same research group had reported a year before [58] that a peripheral nerve lesion activates microglia selectively and focally in the region of the mesolimbic dopaminergic system, affecting the entire reward circuit [59-61].

Targeting microglia as a therapeutic strategy in treating neuropathic pain

Since the involvement of microglia in the development and maintenance of neuropathic pain has been confirmed, the investigation of new drugs able to modify the function of immune cells of the CNS is a priority and a challenge for researchers [62]. There is an urgent need for new, safe, and effective drugs for neuropathic pain, which could constitute a combination therapy that targets both neural (neurons) and non-neural (microglia and astrocytes) cells. Since microglial cells are immune cells of the CNS, they would be suppressed using anti-inflammatory and immunosuppressive drugs. Both drug categories have been used to reduce microglia activation, control inflammation and neuroinflammation, and improve the clinical symptoms of neuropathic pain [63]. Table 1 summarizes the immunomodulatory drugs that target microglia and potentially may be used to treat neuropathic pain, according to animal and experimental studies.

**Table 1:** Anti-inflammatory and immunomodulatory drugs that target microglial cells MAP: mitogen-activated protein TNF: tumor necrosis factor CNS: central nervous system NMDA: N-methyl-D-aspartate IL: interleukin COX: cyclooxygenase PPAR: peroxisome proliferator-activated receptor

Drug/substance	Mechanism of action	Effect on models of neuropathic pain	References
Minocycline	Tetracycline. Partly immunosuppressive because it inhibits the p38 MAP signaling pathway and cellular growth	Prevents the development of neuropathic pain (hyperalgesia and allodynia) by inhibiting microglial cells when given before the nerve injury	[1,51,59,64-70]
Pentoxifylline	Non-selective phosphodiesterase inhibitor. Potent TNF-α inhibitor	Reduces mechanical allodynia by suppressing the activation of microglia and astrocytes. It is often accompanied by a decrease in pro-inflammatory cytokines	[22,27,65,69,71,72]
Methotrexate	Reduces the concentration of folic acid, de novo inhibits purines and synthesis of thymidylic acid	Inhibits the activation and proliferation of microglia. Effective when administered after nerve damage	[73]
Nucleotide receptor antagonists	The activation of P2X & P2Y purinergic receptors modulates the activity of peripheral immune cells and microglial cells in the CNS	Inhibit the activation of peripheral macrophages and spinal microglia	[74]
P38 MAP kinase inhibitors and ketamine	Inhibit important signaling pathways of microglia. Specifically, ketamine inhibits NMDA receptors	Reduce tactile allodynia. Effective when administered before nerve damage. Use of ketamine in neuropathic pain.	[50,51,75-77]
Neutralizing antibodies and receptor – trapping strategies	Substances that control the synthesis of cytokines and act against IL1, IL6, IL10, & TNF	They reduce the biological effects of pro-inflammatory cytokines. IL10 also has anti-inflammatory action	[69]
Complement inhibitors	They prevent the activation of the complement expressed by microglia. They block the classical pathway of the complement system	Inhibit complement activation pathways, including the C5 pathway, which acts as a chemoattractant in the posterior horn of the spinal cord	[69,78]
Cannabinoids	Activate CB1 & CB2 cannabinoid receptors, which are particularly expressed in microglia	CB2 receptors regulate the migration and proliferation of immune cells. CB2 agonists reduce mechanical allodynia	[79,80]
Nonsteroidal anti-inflammatory drugs	Inhibition of prostaglandin PGE2 of microglial cells, inhibition of COX, inactivation of microglial via PPAR-γ pathway	Anti-inflammatory action. Inhibition of activated microglia. Inhibition of β-amyloid production in the brain, inhibition of β and γ-secretase. Reduction of proliferation	[13,79,81-84]

## Conclusions

Peripheral nerve damage and peripheral inflammation are known causes of chronic pain. In situations of sensitization, prolonged activation of the primary sensory nerves in the periphery is followed by increased activity of the terminal nerve cells in the posterior horn of the spinal cord. However, hyperactivity of the nerves in the area has the effect of activating non-nerve cells (immune cells) at the site of the lesion (partial activation). Microglial cells are thus involved in nervous pathology (disease or injury) dynamically; they proliferate, change morphologically, and secrete substances that make local synapses even more sensitive. Therefore, a condition of positive stimulus feedback is established, leading to chronicity. Activation of microglia is more likely after nerve damage than after inflammation. They are found along the entire length of the somatosensory pathway, where immune cells are present at each synapsis. Microglia participate in all processes of the mechanism that produces chronic neuropathic pain (gliopathy). The interaction between neurons and microglial cells is considered critical for chronic pain and inflammation. The disruption of the two-way relationship between neurons and immune cells is associated not only with chronic neuropathic pain but is considered the pathogenetic factor for a number of inflammatory diseases of the peripheral and central nervous system, which are called neurodegenerative diseases.

Concluding, the somatosensory system is a key regulator of the innate immune response of organisms. To achieve this, it interacts and cooperates with the cells of the immune system, in particular with microglia. Understanding this communication, which we have tried to describe in detail in this review, will unfold new cellular mechanisms, lead research into interesting biochemical molecular pathways, and turn medical thought to a more holistic view of the organic systems involved in chronic pain (neuro-immune-endocrine coupling).
